# Hybrid Instantaneous Wave-Free Ratio–Fractional Flow Reserve versus Fractional Flow Reserve in the Real World

**DOI:** 10.3389/fcvm.2017.00035

**Published:** 2017-05-30

**Authors:** Kara Shuttleworth, Kristina Smith, Jonathan Watt, Jamie A. L. Smith, Stephen J. Leslie

**Affiliations:** ^1^Cardiac Unit, Raigmore Hospital, Inverness, UK; ^2^Department of Diabetes and Cardiovascular Science, The Centre for Health Science, University of the Highlands and Islands, The Centre for Health Science, Inverness, UK

**Keywords:** coronary stenosis, functional assessment, angiography, instantaneous wave-free ratio, fractional flow reserve

## Abstract

**Background:**

The instantaneous wave-free ratio (iFR) is a novel method to assess the ischemic potential of coronary artery stenoses. Clinical trial data have shown that iFR has acceptable diagnostic agreement with fractional flow reserve (FFR), the reference standard for the functional assessment of coronary stenoses. This study compares iFR measurements with FFR measurements in a real world, single-center setting.

**Methods and results:**

Instantaneous wave-free ratio and FFR were measured in 50 coronary artery lesions in 42 patients, with FFR ≤ 0.8 classified as functionally significant. An iFR-only technique, using a treatment cut-off value, iFR ≤ 0.89, provided a classification agreement of 84% with FFR ≤ 0.80. Use of a hybrid iFR–FFR technique, incorporating FFR measurement for lesions within the iFR gray zone of 0.86–0.93, would improve classification agreement with FFR to 94%, with diagnosis achieved without the need for hyperemia in 57% patients.

**Conclusion:**

This study in a real-world setting demonstrated good classification agreement between iFR and FFR. Use of a hybrid iFR–FFR technique would achieve high diagnostic accuracy while minimizing adenosine use, compared with routine FFR.

## Key Questions

### What is Already Known about the Subject?

- Coronary angiography alone is relatively poor at assessing the functional significance of coronary lesions.
- Fractional flow reserve (FFR) is an established method to guide coronary intervention but requires routine adenosine administration.
- Instantaneous wave-free ratio (iFR) in trial populations may help diagnosis without the use of adenosine.
- The impact of iFR in a non-trial setting is less certain.

### What Does This Study Add?

A hybrid approach in a non-trial setting will allow most patients to avoid FFR and adenosine in about half of patients.

### How Might This Impact on Clinical Practice?

This study may positively influence the use of iFR to guide coronary intervention, simplifying the procedures in the cardiac catheterization laboratory.

## Introduction

Over the past two decades, randomized studies have demonstrated that routine measurement of FFR is superior to angiographic assessment alone for improving outcome in patients undergoing percutaneous coronary intervention (PCI) (1, 2). This has led to the widespread adoption of FFR to evaluate the functional significance of coronary lesions and guide revascularization in clinical practice (3).

The theory behind FFR is underpinned by the linear relationship between coronary pressure and flow under conditions of constant minimal coronary microvascular resistance (4, 5). FFR is calculated from measurements taken during pharmacologically induced hyperemia (6), most commonly achieved using intravenous (i.v.) adenosine. However, the use of i.v. adenosine can cause patient discomfort, is not suitable for patients with asthma, and adds time and cost to the procedure. Although intracoronary adenosine can improve patient comfort, compared with i.v. adenosine (7), the induction of maximal hyperemia and confirmation of FFR can remain difficult to interpret (8).

The recent development of iFR allows the physiological significance of a coronary stenosis to be assessed without the requirement to induce hyperemia. iFR is calculated by measuring the resting pressure gradient across a coronary stenosis during the diastolic wave-free period within a single cardiac cycle, when coronary resistance is low and stable (8). Therefore, the functional assessment of coronary stenoses using iFR is quicker compared with FFR and avoids the need to administer adenosine.

It is known that the figures generated by iFR and FFR are not inter-changeable. However, the relationship between iFR and FFR has been extensively evaluated (9–12), with a diagnostic agreement of 80–90%, depending on the severity of the lesion being assessed (9, 11). Studies suggest that an FFR cut-off of ≤ 0.80 is comparable to an iFR cut-off of ≤ 0.89 (9). When compared with independent measurements of ischemia, iFR and FFR have demonstrated comparable diagnostic accuracy (13, 14).

Despite these studies, the reliability of iFR has undergone significant scrutiny and debate in the literature since it was first introduced (15–17). To address some concerns regarding differences between iFR and FFR, a hybrid iFR–FFR decision-making strategy has been proposed by introducing the concept of an iFR “gray zone” between 0.86 and 0.93 (14) (Table 1). However, most of this work has been undertaken in the setting of a clinical trial. It is also worth noting that while iFR uses FFR as a reference standard, FFR itself has its own limitations and gray zones. Trials have shown that FFR has a gray zone between 0.75 and 0.85 (18). A current trial is investigating the best method for investigating those arteries which fall into the gray-zone FFR (http://www.hra.nhs.uk/news/research-summaries/the-gray-zone-ffr-study-gzffr-study/). The gold standard for determining ischemia remains unproven.

**Table 1:** **Fractional flow reserve (FFR) and instantaneous wave-free ratio (iFR) “cut-off” and “hybrid” strategy**.

Treatment decision	FFR only	iFR only	iFR hybrid
Revascularize	≤0.8	≤0.89	<0.86
Gray zone	NA	NA	0.86–0.93 (proceed to FFR)
Defer treatment	>0.8	>0.89	>0.93

This current study looked at the real-life use of iFR and FFR and assessed the diagnostic agreement between iFR and FFR using both the hybrid strategy and absolute cut-offs.

## Materials and Methods

### Patients and Setting

This retrospective study was undertaken at a regional cardiac center in the North of Scotland with a single cardiac catheterization laboratory between December 2014 and June 2015. A convenience sample of patients undergoing elective or *ad hoc* physiological assessment of a coronary artery stenosis of intermediate angiographic severity was included. Patients were excluded if adenosine could not be used.

### iFR and FFR Measurement

Cardiac catheterization was performed by either a radial or femoral approach (at the discretion of the operator). A 0.014-inch pressure sensor-tipped wire (PrimeWire Prestige, Volcano Corporation, San Diego, CA, USA) was positioned with the sensor at the distal tip of the guiding catheter. Normalization of the pressure trace was performed before the wire was advanced distal to the target lesion. iFR was initially recorded using the integrated Volcano CORE system, with FFR subsequently recorded during peripherally administered i.v. adenosine-induced stable hyperemia. Adenosine was infused at a rate of 8.4 mg/kg per hour. Clinical decisions were left to the discretion of the operator.

### Diagnostic Strategies and Definitions

Diagnostic strategies were hypothetically applied to the results post procedure as shown in Table 1. When testing the iFR-only technique, an iFR ≤ 0.89 was classified as functionally significant and, therefore, suitable for PCI, lesions with iFR > 0.89 were deferred for treatment. For the FFR approach, a treatment cut-off of ≤ 0.8 was applied. When testing the iFR–FFR hybrid technique, an iFR gray zone of 0.86–0.93 was applied, between these values FFR would be measured.

### Data Collection and Statistical Analysis

The results of the iFR, FFR, and target artery were recorded. Data were entered into a database. Summary statistics were used for comparing a patient’s true disease (as indicated by FFR) to the diagnostic measurement obtained by iFR. Classification of vessels was assessed via a 4 × 4 contingency table for true and false positives and true and false negatives (Microsoft Excel version 2007).

### Ethics

This was a service evaluation of routinely collected data with no contact made with the patients and therefore ethical approval was not required. Data collected was anonymized using a unique study number with only the senior clinician aware of the patient details.

## Results

The relationship between iFR and FFR was evaluated in 50 lesions in 42 patients, 31 (74%) were male, with a mean age of 63 ± 9 years. The coronary lesions assessed included 21 (42%) in the left anterior descending (LAD), 16 (32%) in the right coronary artery (RCA), and eight (16%) in the circumflex artery (Cx).

**Figure 1:**
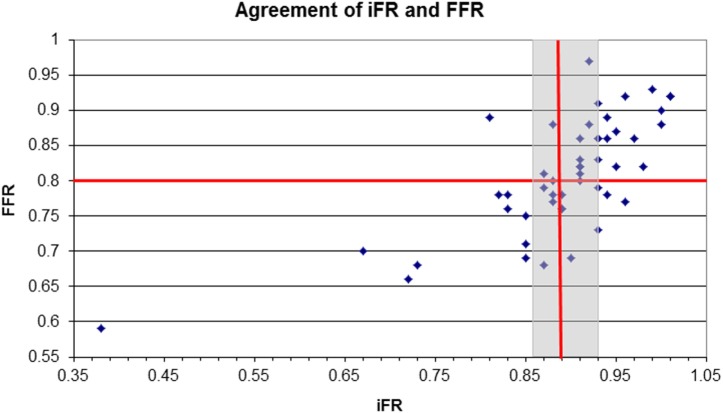
**Scatter plot of instantaneous wave-free ratio (iFR) and fractional flow reserve (FFR) measurements for individual lesions**. The treatment cut-off points of iFR ≤ 0.89 and FFR ≤ 0.8 are marked with red lines. The gray zone for iFR of 0.86–0.89 is shown by the gray box.

Analysis of an iFR-only strategy, with a treatment cut-off of ≤ 0.89 showed a diagnostic classification agreement of 84%, when compared with the FFR-only strategy (treatment cut-off of ≤ 0.8). iFR had a sensitivity of 82%, a specificity of 83%, a positive predictive value of 88%, and a negative predictive value of 75%. PCI would have been deferred in 29 lesions (58%) based on iFR measurements and 26 lesions (52%) based on FFR. Using the hybrid iFR–FFR strategy, 47 lesions (94%) were accurately classified compared to FFR (Figure 1), with PCI being deferred in 27 lesions (54%). This hybrid strategy would have also resulted in 25 (50%) lesions being diagnostically classified without the use of adenosine and resulted in 24 (57%) patients not receiving adenosine.

In three (6%) lesions, there was a disagreement of classification between iFR and FFR (Figure 1). The one lesion where iFR was < 0.86 and FFR was ≥ 0.8 was located in the LAD. The remaining two lesions where iFR was > 0.93 but FFR ≤ 0.8 were located in the RCA.

## Discussion

This study investigates the diagnostic utility of iFR and FFR in a real-world population in a single center. iFR and FFR showed good agreement when using established cut-offs but improved agreement when the hybrid approach was employed. The potential to perform functional assessment of coronary stenoses without the administration of adenosine has many potential advantages, including reduced procedure times, improved patient comfort, and reduced procedural costs. However, despite clear advantages, the use of iFR is still under much scrutiny and debate.

This single-center study confirms a good agreement between the iFR of ≤ 0.89 and the current reference standard FFR ≤ 0.80, which has previously been suggested in the ADVISE and RESOLVE trials (10, 13). A classification agreement of 84% between iFR and FFR is consistent with off-line data from the ADVISE registry (10). Unsurprisingly, classification agreement was limited close to the established cut-off values (in the gray zone). Therefore, the recently recommended hybrid iFR–FFR approach was applied and this improved the classification agreement to 94%. The application of the hybrid iFR–FFR method would also have resulted in adenosine-free lesion classification for the majority of our patients. iFR data are limited in that there have been no studies reporting the clinical outcomes of patients undergoing iFR-guided treatment. The true significance of any clinical difference when applying this hybrid approach has been explored in the DEFINE-FLAIR study, where clinical outcomes in 2500 patients were compared using iFR-guided treatment versus FFR-only-guided treatment (19). Another study expected to provide much needed clarification on whether the level of disagreement between iFR and FFR is clinically relevant is the iFR-SWEDEHEART trial, with results expected imminently (20).

Applying the historical FFR cut-off of ≤ 0.75 (as used in the DEFER study) to our hybrid iFR strategy led to classification disagreement in only one lesion, located in the LAD which had an iFR < 0.86 but FFR > 0.8. Similar findings were reported in a real-world experience described by Harle et al. (21).

Following the findings of this study, we think that the iFR–FFR hybrid approach is a valuable method of implementing iFR in real clinical practice, which would reduce procedural times and costs. Furthermore, as the iFR hybrid approach is less time-consuming than routine FFR, frequent use of invasive functional coronary assessment, which remains underutilized, may be more easily achievable (22). The recently completed DEFINE-FLAIR study confirmed non-inferiority of iFR alone versus FFR with a significant reduction in periprocedural patient discomfort with iFR (23) which would seem to suggest that better patient outcomes can be achieved with iFR alone than with the iFR–FFR hybrid approach used in our study.

However, given the recent early discontinuation of the FUTURE trial evaluating patients with multivessel coronary disease (due to excess deaths in the FFR group) clinicians should be cautious in the use of iFR or FFR in this specific patient group (https://clinicaltrials.gov/ct2/show/NCT01881555).

Further research may refine appropriate cut-off values for both methods, although one might argue that definite cut-off values are not biologically sensible and the use of a gray zone, where clinical decision-making can be individualized, is more realistic.

### Limitations

The relatively small number of patients limits the power of these observations, as with the fact that this was a single-center trial.

## Conclusion

In a real-world clinical setting, we demonstrated good agreement between classification of lesions using iFR and FFR, with improved agreement when applying a hybrid iFR approach, using FFR only for lesions within the iFR gray zone. This hybrid strategy (if implemented) would reduce the number of patients requiring the administration of adenosine to functionally assess stenoses. iFR appears to be a promising diagnostic tool, given the importance of functional coronary assessment.

## Ethics Statement

This was a service evaluation of routinely collected data with no contact made with the patients and, therefore, ethical approval was not required. Data collected were anonymized using a unique study number with only the senior clinician aware of the patient details.

## Author Contributions

Concept and design: KSmith, JS, and SL. Data analysis: KShuttleworth, KSmith, JW, and SL. Interpretation of the data, drafting the manuscript, final approval of the submitted version, and agreement to be accountable: KShuttleworth, KSmith, JW, JS, and SL.

## Conflict of Interest Statement

The authors declare that the research was conducted in the absence of any commercial or financial relationships that could be construed as a potential conflict of interest. The reviewer NB and handling Editor declared their shared affiliation, and the handling Editor states that the process nevertheless met the standards of a fair and objective review.

## References

[1] Tonino PA De Bruyne B Pijls NH Siebert U Ikeno F van’ t Veer M Fractional flow reserve versus angiography for guiding percutaneous coronary intervention. N Engl J Med (2009) 360:213–24. 10.1056/NEJMoa0807611 19144937

[2] Bech GJ De Bruyne B Pijls NH de Muinck ED Hoorntje JC Escaned J Fractional flow reserve to determine the appropriateness of angioplasty in moderate coronary stenosis: a randomized trial. Circulation (2001) 103:2928–34. 10.1161/01.CIR.103.24.2928 11413082

[3] Tonino PA Fearon WF De Bruyne B Oldroyd KG Leesar MA Ver Lee PN Angiographic versus functional severity of coronary artery stenoses in the FAME study: fractional flow reserve versus angiography in multivessel evaluation. J Am Coll Cardiol (2010) 55(25):2816–21. 10.1016/j.jacc.2009.11.096 20579537

[4] Kern MJ An adenosine-independent index of stenosis severity from coronary wave-intensity analysis: a new paradigm in coronary physiology for the cath lab? J Am Coll Cardiol (2012) 59(15):1403–5. 10.1016/j.jacc.2011.11.006 22154777

[5] Spaan JA Piek JJ Hoffman JI Siebes M. Physiological basis of clinically used coronary hemodynamic indices. Circulation (2006) 113(3):446–55. 10.1161/CIRCULATIONAHA.105.587196 16432075

[6] Pijls NH Van Gelder B Van der Voort P Peels K Bracke FA Bonnier HJ Fractional flow reserve. A useful index to evaluate the influence of an epicardial coronary stenosis on myocardial blood flow. Circulation (1995) 92(11):3183–93. 10.1161/01.CIR.92.11.3183 7586302

[7] Schlundt C Bietau C Klinghammer L Wiedemann R Rittger H Ludwig J Comparison of intracoronary versus intravenous administration of adenosine for measurement of coronary fractional flow reserve. Circulation (2015) 8(5):e001781. 10.1161/CIRCINTERVENTIONS.114.001781 25908694

[8] Tarkin JM Nijjer S Sen S Petraco R Echavarria-Pinto M Asress KN Hemodynamic response to intravenous adenosine and its effect on the fractional flow reserve assessment, results of the adenosine for the functional evaluation of coronary stenosis severity (AFFECTS) study. Circ Cardiovasc Interv (2013) 6:654–61. 10.1161/CIRCINTERVENTIONS.113.000591 24254709

[9] Sen S Escaned J Malik IS Mikhail GW Foale RA Mila R Development and validation of a new adenosine-independent index of stenosis severity from coronary wave-intensity analysis: results of the ADVISE (ADenosine Vasodilator Independent Stenosis Evaluation) study. J Am Coll Cardiol (2012) 59:1392–402. 10.1016/j.jacc.2011.11.003 22154731

[10] Petraco R Escaned J Sen S Nijjer S Asrress KN Echavarria-Pinto M Classification performance of instantaneous wave–free ratio (iFR) and fractional flow reserve in a clinical population of intermediate coronary stenoses: results of the ADVISE registry. EuroIntervention (2013) 9(1):91–101. 10.4244/EIJV9I1A14 22917666

[11] Park JJ Petraco R Nam CW Doh JH Davies J Escaned J Clinical validation of the resting pressure parameters in the assessment of functionally significant coronary stenosis; results of an independent, blinded comparison with fractional flow reserve. Int J Cardiol (2013) 168(4):4070–5. 10.1016/j.ijcard.2013.07.030 23890849

[12] Jeremias A Maehara A Genereux P Asrress KN Berry C De Bruyne B Multicenter core laboratory comparison of the instantaneous wave–free ratio and resting P/P with fractional flow reserve: the RESOLVE study. J Am Coll Cardiol (2013) 63(13):1253–61. 10.1016/j.jacc.2013.09.060 24211503

[13] Sen S Asrress KN Nijjer S Petraco R Malik IS Foale RA Diagnostic classification of the instantaneous wave-free ratio is equivalent to fractional flow reserve and is not improved with adenosine administration. Results of CLARIFY (Classification Accuracy of Pressure-Only Ratios Against Indices Using Flow Study). J Am Coll Cardiol (2013) 61:1409–20. 10.1016/j.jacc.2013.01.034 23500218

[14] Petraco R van de Hoef TP Nijjer S Sen S van Lavieren MA Foale RA Baseline instantaneous wave-free ratio as a pressure-only estimation of underlying coronary flow reserve: results of the JUSTIFY-CFR Study (joined coronary pressure and flow analysis to determine diagnostic characteristics of basal and hyperemic indices of functional lesion severity-coronary flow reserve). Circ Cardiovasc Interv (2014) 7:492–502. 10.1161/CIRCINTERVENTIONS.113.000926 24987048

[15] Finet G Rioufol G A new adenosine-independent index of stenosis severity: why would one assess a coronary stenosis differently? J Am Coll Cardiol (2012) 59(21):1915 10.1016/j.jacc.2012.01.048 22595414

[16] Pijls NH Van ’t Veer M Oldroyd KG Berry C Fearon WF Kala P Instantaneous wave-free ratio or fractional flow reserve without hyperemia: novelty or nonsense? J Am Coll Cardiol (2012) 59(21):1916–7. 10.1016/j.jacc.2012.01.049 22595415

[17] Rudzinski W Waller AH Kaluski E Instantaneous wave-free ratio and fractional flow reserve: close, but not close enough! J Am Coll Cardiol (2012) 59(21):1915–6. 10.1016/j.jacc.2012.01.047 22595413

[18] Petraco R Park JJ Sen S Nijjer SS Malik IS Echavarría-Pinto M Hybrid iFR–FFR decision-making strategy: implications for enhancing universal adoption of physiology-guided coronary revascularisation. EuroIntervention (2013) 8(10):1157–65. 10.4244/EIJV8I10A179 23256988

[19] Functional Lesion Assessment of Intermediate Stenosis to Guide Revascularisation (DEFINE-FLAIR). NCT02053038. Avaliable from: https://www.clinicaltrials.gov/ct2/show/NCT02053038

[20] Gotberg M Christianses E Gumundsdottir I Sandhall L Omerovic E James SK Instantaneous wave free ratio versus fractional flow reserve guided intervention (iFR-SWEDEHEART): rationale and design of a multicenter, prospective, registry-based randomized clinical trial. Am Herat J (2015) 150(5):945–50. 10.1016/j.ahj.2015.07.031 26542503

[21] Harle T Waldemar B Meye S Elsasser A Comparison of instantaneous wave-free ratio (iFR) and fractional flow reserve (FFR) – first real world experience. Int J Cardiol (2015) 199:1–7. 10.1016/j.ijcard.2015.07.003 26179896

[22] Townend JN Ludman PF Doshi SN Khan H Calvert PA Assessing flow limitation in stable angina, has simple coronary angiography had its day? BMJ (2016) 355:i5534 10.1136/bmj.i5534 27765788

[23] Davies JE Sen S Dehbi HM Al-Lamee R Petraco R Nijjer SS Use of the instantaneous wave-free ratio or fractional flow reserve in PCI. N Engl J Med (2017) 376:1824–34. 10.1056/NEJMoa1700445 28317458

